# End-Stage Cancer Patients Diagnosed with a Femoral Pathological Fracture on Admission to Palliative Care Units

**DOI:** 10.1089/pmr.2021.0052

**Published:** 2021-12-09

**Authors:** Hironobu Kanazawa

**Affiliations:** Department of Palliative Care Medicine, Sagara Hospital, Kagoshima, Japan.

**Keywords:** delirium, end-of-life cancer patients, family grief, femoral pathological fracture, medical staff, palliative care unit

## Abstract

**Background:**

Femoral pathological fractures (PFs) due to bone metastasis result in exacerbation of pain, gait disturbance, and reduced general condition. Surgery may be considered depending on the situation, but is not suggested often, and treatment is difficult toward the end of life.

**Objective:**

Terminal cancer patients with a femoral PF admitted to a palliative care unit (PCU) were retrospectively evaluated.

**Measurement:**

Seven cancer patients diagnosed with a femoral PF at a PCU on admission, in Japan, were examined for clinical background, physical symptoms, and psychiatric symptoms. In addition, the responses of the patients' families and medical staff were examined. This study was approved by the ethics board of our hospital.

**Results:**

A total of 28.6% of patients were hospitalized from home, and the trigger for PF could not be confirmed in 85.7% of patients. In all cases, surgery was not recommended, given the poor prognosis. Opioid drugs were used for pain in all patients, and 85.7% of patients were able to relieve their symptoms. Delirium was observed in 71.4% of cases, and treatment with antipsychotics was required in all cases. Family grief also emerged as a problem, and the staff was burdened; hence, we addressed this at the death conference.

**Conclusions:**

Even for femoral PFs in cancer patients with a limited prognosis, it is necessary to perform tests and control pain. In addition, it is important to support the mental distress of patients and their families in a short period; medical staff should be trained to support the families after the patients' death.

## Introduction

Bone is a part of distant metastases at a high frequency,^1–3^ and bone-related events such as pain, spinal cord compression, pathological fractures (PFs), and hypercalcemia develop in patients with advanced cancer. Therefore, these are considered poor prognosis factors because the general condition and quality of life (QOL) are significantly reduced.^4,5^ PF occurs due to a slight external force such as load or bruise in addition to a decrease in bone strength, caused by primary and metastatic bone tumors, osteoporosis, and bone system diseases.^6^

If PF occurs in the femur due to bone metastasis, physical distress occurs due to increased pain, loss of motor function such as difficulty in walking, and the deterioration of one's general condition due to the progression of carcinoma.^3^ Regarding treatment, surgery or radiation can be performed, but toward the end of life (EOL), treatment becomes difficult. In addition, at the terminal stage of cancer, psychological distress such as delirium, anxiety, and depression becomes more pronounced, and the inability to move as one wishes due to PF is a risk factor.^7,8^

This makes the situation painful for the family, the patient,^9^ and the medical staff; particularly, those engaged in palliative care units (PCUs) are pained, helpless, and defeated by thinking that the patient may not die peacefully. By holding discussions on EOL care in death conferences (DCs),^10,11^ medical staff in PCUs can understand each other, deepen trust relationships, and use this opportunity to think about caring for patients and stress reduction.

Previous studies have not examined the treatment plans and mental status of patients with PF in the terminal stage of cancer. Furthermore, the viewpoints of families and medical staff have not yet been studied. In this study, I retrospectively examined a clinical study of patients who had developed femoral PF at the time of admission, and how this affected their families and medical staff through the use of DC sheets. Thus, this report is useful not only for PCU staff but also for medical and nursing staff involved in cancer care.

## Materials and Methods

I retrospectively examined seven EOL-stage cancer patients diagnosed with femoral PF in the PCU on admission between January 2018 and December 2020 in Kagoshima, Japan. PF is defined as the “breaking of a bone by a slight external force that would not normally cause a fracture with metastatic bone tumors confirmed by image findings.” The conditions before admission were confirmed from the patient's referral document and imaging tests (X-ray, CT, MRI, bone scintigraphy, and/or fluorodeoxyglucose-positron emission tomography) of the hospital before the transfer as all cases were referred from other hospitals.

The fracture status after hospitalization of all patients was assessed by radiography on the bed because it was difficult to move them. The situation of the femoral PF, treatment for pain, orthopedic consultation, psychiatric symptoms, and prognosis were studied. Since it was difficult to subjectively evaluate pain treatment in some cases, the Japanese version Support Team Assessment Schedule (STAS-J) symptom version was used for objective evaluation.^12^

Using this scale, the medical staff objectively evaluated the patients' pain on a 5-point scale (0 = no symptoms; 1 = an occasional or single minor symptom that the patient does not think needs to be resolved; 2 = moderate distress, occasional bad days, and symptoms limiting some activities within the extent of the disease; 3 = a severe symptom that is often present and greatly affects the patient's activities and concentration; and 4 = a severe and continuous overwhelming symptom that prevents the patient from thinking of other things). A score of 2 or more was judged to be painful in this study.

Benefit from treatment was defined as a decrease in the score, when the symptoms were alleviated after the treatment and were the strongest before the intervention.^34^ I evaluated the mental distress of patients and their families by enquiring about the deceased patient's PF, and the PCU staff looked back on treatment and care by holding a DC after the death of each patient. The DC sheet of our facility was investigated retrospectively and analyzed with reference to the report by Kako et al.^10^ Contents of the DC sheet consisted of case presentation, retrospectively analyzing case problems, solving problems and positive evaluation, things that made them think and learn, and a summary of cases and clarification of future issues.

Since patients had already died and this study was a noninvasive observational trial, I used an opt-out method and did not require written or oral informed consent from the patients or their families. This study was approved by the ethics board of our hospital, and was conducted in accordance with the principles of the Declaration of Helsinki.

Data analysis using descriptive statistics was performed in Microsoft Excel 2019 (Redmond, WA, USA).

## Results

The clinical background of the patients on admission is shown in Table 1. Five female patients (71.4%) had an average age of 66.9 ± 13.8 years at the time of hospitalization, and the most common primary tumor was lung cancer. Six patients (85.7%) had a history of cancer drug therapy. Two patients (28.6%) were urgently hospitalized from home, and five cases (71.4%) were transferred from another hospital. The Eastern Cooperative Oncology Group Performance Status (ECOG PS) was 1 in one case (14.3%), 3 in two cases (28.6%), and 4 in four cases (57.1%), with an average of 3.4 ± 0.7. Before admission, femoral metastasis was noted in one case (14.3%), and three cases (42.9%) were not examined for it.

**Table 1.** Clinical Characteristics of the Participants

Characteristic	
Age range (years)	46–90
Average age (years ± SD)	66.9 ± 13.8
Gender	
Female	5 (71.4)
Male	2 (28.6)
Primary lesion	
Pulmonary	3 (42.9)
Cervix	1 (14.3)
Kidney	1 (14.3)
Pancreas	1 (14.3)
Bile duct	1 (14.3)
Metastatic lesions on admission	
OSS	4 (57.1)
PUL	3 (42.9)
BRA	2 (28.6)
Prehospital location	
Hospital	5 (71.4)
Home	2 (28.6)
Cancer treatment history	
Drug	6 (85.7)
None	1 (14.3)
PS at the time of admission	3.4 ± 0.7
2	1 (14.3)
3	2 (28.6)
4	4 (57.1)

Values are mean ± SD, range, or *n* (%).

BRA, brain; OSS, bone; PS, performance status; PUL, pulmonary.

Table 2 shows the status of PF at the time of admission. All patients had pain at the femoral PF site, and so, the diagnosis was made by the roentgen performed at the bedside to increase pain on movement and poor PS. Fall episodes were confirmed in only one case (14.3%), and the trigger for PF could not be confirmed in the other cases (85.7%). The femoral PF of the distal site was one case (14.3%) and the proximal portion (neck or trochanteric) in six patients (85.7%).

**Table 2.** Fracture Condition at Admission

Status	n (%)
Fracture location	
Right/left	5 (71.4)/2 (28.6)
Site	
Neck	4 (57.1)
Trochanteric	2 (28.6)
Distal	1 (14.3)
Condition pointed out before hospitalization	
Yes	1 (14.3)
No	3 (42.9)
Not examined	3 (42.9)
Treatment history of fracture site	
No	6 (85.7)
Radiation	1 (14.3)
Causes of fracture	
Unknown	6 (85.7)
Fall	1 (14.3)
Tissue classification	
Osteolytic	7 (100)
Symptoms of fracture site	
Pain	7 (100)
Swelling	5 (71.4)
Heat sensation	2 (28.6)
Hypercalcemia	2 (28.6)
Other symptoms	
Fatigue	6 (85.7)
Dyspnea	3 (42.9)
Nausea	1 (14.3)
Orthopedic consultation	3 (42.9)

Hypercalcemia was observed in two cases (28.6%) due to complications. Three patients (42.9%) consulted orthopedists at another hospital, but surgery was not recommended due to poor prognosis, and four patients (57.1%) were judged to have difficulty undergoing surgery considering the prognosis and general condition at our PCU. Considering the prognosis in all cases, the patients were rested on the bed without traction. Regarding drug therapy for pain, opioid analgesics were used for the first time for PF pain in three cases (42.9%); all cases, including those who were originally treated with oral medication, were finally changed to continuous injection. Two cases (28.6%) were given lidocaine and one case (14.3%) received ketamine as an adjuvant analgesic (Table 3). Although the therapeutic effect on pain could not be evaluated subjectively in some cases, it was considered to be effective in six cases (85.7%) by objective assessment using the STAS-J symptom version. Delirium was observed in six cases (85.7%) between admission, and three cases (42.9%), including two cases (28.6%) who were urgently hospitalized from home, were newly diagnosed during hospitalization. Treatment with antipsychotic agents was required in all cases. The average survival time after hospitalization in PCU was 22.1 ± 20.6 days. In the survival prediction by the palliative prognostic index (PPI), established cutoff points set by Morita et al.,^21^ five cases (71.4%) had a score >6, indicating that survival would be less than three weeks, of which four cases had prognoses of less than three weeks, and the average survival time was 11.0 ± 5.7 days (Table 3).

**Table 3.** Summary of Posthospital Courses

Opioid	
Prehospitalization	
Used/PO	4 (57.1)
Not used	3 (42.9)
Posthospitalization	
Used/IV or SC	7 (100)
Adjuvant analgesics	
Prehospitalization	
Not used	7 (100)
Posthospitalization	
Ketamine	2 (28.6)
Lidocaine	1 (14.3)
Not used	4 (57.1)
Effects	
NRS	
Effective	3 (42.9)
Undeterminable	4 (57.1)
STAS-J	
Effective	6 (85.7)
Noneffective	1 (14.3)
Delirium	
On admission	3 (42.9)
After admission	6 (85.7)
Sedation	
Continuous	1 (14.3)
Intermittent	1 (14.3)
Hospitalization	
Length (days)	5–68
Average (days ± SD)	22.1 ± 20.6
PPI	
PPI on admission	10.0 ± 4.1
≤3.5	1 (14.3)
≤4, ≤6	1 (14.3)
≤6.5	5 (71.4)
Family grief	6 (85.7)
Strongest grief	
Spouse	3 (42.9)
Child	2 (28.6)
Parents	1 (14.3)
Main causes of grief	
Delirium	3 (42.9)
Long bed rest	2 (28.6)
Short prognosis	1 (14.3)

Values are mean ± SD, range, or *n* (%).

PO, oral administration; IV, intravenous; SC, subcutaneous; NRS, Numeric Rating Scale; UD, undeterminable; STAS-J, Japanese-version Support Team Assessment Schedule; PPI, Palliative Prognostic Index.

Confirmed family grief was seen in six cases, most often in spouses, depending on family composition. The content of the grief was not only a short prognosis and delirium at the EOL, but also bed rest due to PF for a long time, which should not have existed originally (Table 3). At the DC held by medical staff after the patient's death, the most important problems highlighted were supporting the family as a care target rather than pain relief and the difficulty of responding to thoughts of the patient him/herself (Table 4).

**Table 4.** Content Analysis of Death Conferences at Our Palliative Care Unit

Category	n (%)
Supporting family members as subjects of care	6 (85.7)
Understanding and cherishing patients' thoughts	2 (28.6)
Reducing symptoms and relieving pain	3 (42.9)
Realizing the importance of communication between medical personnel	2 (28.6)
Becoming anxious due to interaction with patients	3 (42.9)

Multiple answers.

By sharing and understanding each other's feelings and solving them through discussions, I was able to reduce and heal the medical staff's stress. Notably, our PCU has a system to consult with a clinical psychologist when it is difficult to resolve the mental distress of patients and their families. However, it was not utilized in this research because the hospitalization period was short, and the family did not opt for it.

## Discussion

In general, lung, breast, and prostate cancers, and renal cell carcinoma are listed as cancers that often cause bone metastasis.^3,4,13,14^ Although bone metastases do not directly affect life, PF may occur due to the weakening of the bone, not only by a fall but also by a slight external force, and its symptoms are likely to appear especially in the spine, femur, humerus, and pelvis.^3,4^ In addition to pain, performing conventional behaviors may be impossible due to impaired motor functions, such as standing and walking.

Local treatment includes surgery and radiation therapy, with the aim of relieving pain and improving QOL.^15–17^ The indications for surgery are prognosis, ability to tolerate surgery, and possible functional recovery, fixation, and support of fractured parts.^18^ However, in the terminal stage of cancer, aggressive treatment is generally difficult, and it has been reported that systemic and local complications after surgery also occur at a high rate. For this reason, surgery is considered difficult.^19,20^ According to a database report, the mortality rate 30 days after a femoral PF was 2.6%.^20^ Since PF itself is a factor that causes a poor prognosis before surgery, it is necessary to consider the indication, including the general condition and prognosis.

Therefore, prognosis prediction is important for determining the treatment policy; systems such as PPI, palliative prognosis (PaP) score, and the Prognosis in Palliative Care Study (PiPS) predictor model can be used as a reference.^21–23^ In this report, the prognosis prediction in PPI was short and in the terminal stage, and it is less likely that PF itself shortened the prognosis (Table 3). For femoral PF occurring in long bones, there has been an attempt to objectively score preoperative severity judgment using the primary lesion, the presence or absence of visceral metastasis, the number of bone metastases, and PS.^19,24^ These can also be considered prognosis predictors for PF of the femur, as in this report. However, when surgery is not possible and conservative treatment is used, the difficulty of relieving pain, the need for traction, and instructions for bed rest are problems. For the weight loss and muscle weakness caused by cachexia in EOL cancer patients, if femoral PF occurs, pain and loss of motor function cause disuse syndrome and pressure ulcers, resulting in impaired QOL.^25^

In this report, the presence of bone metastasis at the femoral PF site before admission was found in only one case, depending on the time of examination at the previous hospital. As there were no symptoms during the treatment, no regular examination was performed to cover the PF site. Furthermore, although pain was observed before the hospital, examinations were not performed because patients were in the terminal stage before being transferred to our PCU.

Except for one case in which a fall was the cause of the fracture, the obvious trigger was unknown. It was inferred that some external force affected the vulnerability of PF unknowingly, and so, these cases became clear after the transfer by examination. In some cases, such as for the therapeutic strategy, since orthopedists were not available in our hospital, we consulted a specialized hospital and followed the instructions for conservative treatment.

As many cases have a poor prognosis, it is difficult to judge the degree of rest and traction, and the pain induced by body movement needs to be mitigated by carefully changing the position on the bed. As a medication, opioids are the primary drugs for pain caused by femoral PF, and the pain could be relieved by using opioids for the first time or by increasing the dose used in other situations.

In all cases, opioids were eventually changed to continuous injections, and it was considered that the combined use of adjuvant analgesics was effective in alleviating pain, except for patients dying in a very short period. Nerve block was examined in cases where a certain degree of survival could be expected, but it was judged as not feasible because of no system to perform nerve block at our hospital and burden of movement for patients. Inability in determining the strategy due to the absence of orthopedic specialists which caused suffering to patients, was a problem.

Physical pain was observed due to the fracture, but mental distress, such as grief and delirium, was frequently observed. In addition to disease progression, grief is thought to be caused by pain, complications, bed rest, inability to do oneself, and unwillingness to burden the family. Especially in two cases of emergency hospitalization from home, environmental changes due to unexpected sudden hospitalization were also felt as unrealistic, and more grief was observed (Table 4).

The onset and exacerbation of delirium were presumed to include some factors such as terminal-stage cancer, brain metastasis, anemia, and/or hypercalcemia, in addition to new elements of the appearance, enhancement of physical distress caused by PF, drug-induced (opioids, steroids, antipsychotics, etc.), and/or environmental changes such as continuous forced bed rest and hospitalization.^7,26,27^ The femoral fracture itself causes physical and mental distress, which is also considered a risk of delirium,^28^ and interventions have been reported to reduce the incidence of delirium in the elderly.^29^

In this report, delirium was also regarded as requiring drug therapy with antipsychotic drugs, and nondrugs have been reported to reduce the incidence of delirium in the elderly. Alternatively, family grief tended to be seen more strongly when hospitalization was prolonged, and PS was not so bad. In addition to disease progression, unrealistic thoughts such as the inability to move increased the grief for families, and it was expressed as anger.

It is necessary to repeatedly explain the expected progress and the actual changes in the medical condition from an early stage, promote family participation in patient nursing according to needs, treat family members with appreciation,^9,30^ guarantee pain relief to families, and not abandon patients. Although family distress tends to be underestimated, a survey on bereaved families has reported that, alongside patients, they experience a variety of stresses during the course of treatment and are often hesitant to talk about their distress in front of patients.^30^ Therefore, early intervention is effective for stress in the family, and its necessity is supported.^31^

The DC is not specified in hospice care programs outside Japan and is presumed to originate in the Japanese hospice and PCU.^10,11^ The significance of DC, looking back at a deceased patient, is established from clinical experience for this purpose, such as building a relationship of trust among various staff related to PCU, sharing information to learn knowledge, skills, and ideas, putting them into practice, and creating a place to ease burnout and healing from stress.

Although it seems that the morbidity and mortality conference (MMC) is equivalent to DC in the West,^32–34^ MMC has a stronger meaning of research and education aimed at improving the quality and system of medical care and is different from DC in Japan, as it is conducted as a part of clinical practice. As the method and purpose are not uniform, DC itself is difficult to be the subject of research. However, in Japan, it is conducted by trial and error at each facility, including hospitals other than PCU.

At our hospital, we hold conferences about issues and problems, as shown in Table 4, and we could use it as a tool to confirm the support system for patients as a team in the future and to heal each other. As a result, we were able to raise awareness and strengthen cohesion of the entire team.

DC has the advantage of sharing information and strengthening cooperation among medical staff as well as of creating an easy-to-speak environment where positive evaluations can be made at daily conferences. This is important because medical staff can be aware of their respective roles and expect the same from each other in the subsequent support of patients and their families. These results contribute to a new report on the perspectives of patients, their families, and medical staff, and highlight the importance of considering and implementing methods according to the characteristics of each facility to care for EOL cancer patients.

In contrast, this is a retrospective report at a single facility, and the format differs at each facility according to the experience and degree of involvement of each medical staff member with the patient; therefore, there is a limit to generalizing DC.

## Conclusion

EOL cancer patients with a femoral PF at the time of admission were retrospectively analyzed. Pain control can be achieved under poor general conditions and limited prognosis. It should be actively suspected and investigated in cases of unprecedented pain. Alternatively, since mental distress is seen in patients, it is important for medical staff to support patients and their families within a limited time. Medical staff also need to hold DCs to provide high-quality palliative care in the future. To generalize the research on DCs, it is necessary to accumulate data by examining the method at each facility and conducting qualitative research, such as via interviews, to investigate what kind of changes have occurred in the staff.

## Clinical Palliative Care Program

Our PCU is configured as a ward in Sagara Hospital, which is a hospital that specializes in the field of breast cancer and is certified as the only specific area cancer hospital in Japan. Medical treatment and care are given according to the Japanese medical system.

Full-time specialist palliative care staff in the PCU comprised 2 physicians, 20 nurses, and 1 medical clerk; support staff comprised 1 pharmacist, 4 therapists, 1 psychologist, and 3 social workers.

Palliative care is delivered to cancer patients from not only our hospital but also to those referred by other hospitals. Through the referral system, the staff from our center get consultations from other hospitals and, after interviews with patients or their families and conferences by PCU staff, decide whether to provide palliative care to those patients. In addition, there is an emergency hospitalization system for sudden changes in the outpatients' conditions. In case of hospitalization, dedicated staff cover nighttime shifts and provide patients and their families with 24-hour support.

In our PCU, the average number of patients, visits per year, and length of stay are 18.9 patients, 237 patients, and 28.1 days, respectively.

## Abbreviations Used

DC: death conference
EOL: end-of-life
MMC: morbidity and mortality conference
PCU: palliative care unit
PF: pathological fracture
PPI: Palliative Prognostic Index
QOL: quality of life
STAS-J: Japanese-version Support Team Assessment Schedule
